# Development of a mobile laboratory system in hydrogen fuel cell buses and evaluation of the performance for COVID-19 RT-PCR testing

**DOI:** 10.1038/s41598-023-44925-7

**Published:** 2023-10-16

**Authors:** Miho Okude, Kenji Suzuki, Asami Naito, Akio Ebashi, Tomoka Kusama, Junichi Kiyotaki, Yusaku Akashi, Yoshihiko Kiyasu, Yoko Kurihara, Shigeyuki Notake, Masaki Takanashi, Tomokazu Setoyama, Yasushi Kawakami, Hiromichi Suzuki

**Affiliations:** 1 LSI Medience Corporation, 3-30-1 Shimura, Itabashi, Tokyo 174-8555 Japan; 2 Center for Cybernics Research, University of Tsukuba, 1-1-1 Tennodai, TsukubaTsukuba, Ibaraki 305-8573 Japan; 3 Department of Infectious Diseases, University of Tsukuba Hospital, 2-1-1 Amakubo, Tsukuba, Ibaraki 305-8576 Japan; 4 Miroku Medical Laboratory Inc, 659-2 Innai, Saku, Nagano, 384-2201 Japan; 5 Department of Clinical Laboratory, Tsukuba Medical Center Hospital, 1-3-1 Amakubo, Tsukuba, Ibaraki 305-8558 Japan; 6 Department of Infectious Diseases, Faculty of Medicine, University of Tsukuba, 1-1-1 Tennodai, Tsukuba, Ibaraki 305-8575 Japan; 7 Division of Infectious Diseases, Department of Medicine, Tsukuba Medical Center Hospital, 1-3-1 Amakubo Tsukuba, Ibaraki, 305-8558 Japan; 8 Department of Laboratory Medicine, Faculty of Medicine, University of Tsukuba, 1-1-1 Tennodai, Tsukuba, Ibaraki 305-8575 Japan

**Keywords:** Microbiology, Diseases

## Abstract

We designed and developed two new types of hydrogen fuel cell (HFC) buses (motorcoach and minibus) with a mobile laboratory system. Feasibility studies have been performed for mobile laboratory testing, particularly for the laboratory performance of COVID-19 RT-PCR (PCR). We evaluated the driving range capability, PCR sample size capacity, turnaround time (TAT), and analytical performance for the detection of SARS-CoV-2. Saliva samples were used for the current study, and the analytical performance was compared with that of the reference PCR. The estimated driving range and sample size capacity of the HFC and HFC minibus were 432 km and 2847 samples, respectively, for the HFC motorcoach and 313 km and 1949 samples for the HFC minibus. For the TAT, the median time between sample submission and completion of PCR was 86 min for the motorcoach and 76 min for the minibus, and the median time between sample submission and electronic reporting of the result to each visitor was 182 min for the motorcoach and 194 min for the minibus. A secondary analysis of 1574 HFC mobile laboratory testing samples was conducted, and all negative samples were found to be negative by reference PCR. Furthermore, all samples were confirmed to be positive by reference PCR or other molecular examinations.

## Introduction

Automated laboratory testing offers a significant advantage for rapid, accurate, and high-throughput laboratory testing. However, the need for heavy medical devices and electronic power are barriers to their utilization at sample collection sites; thus, samples must be transported to centralized laboratories for laboratory testing. Consequently, it takes days for patients to obtain results. Point-of-care testing (POCT), including rapid antigen testing^1,2^ and POCT-type molecular testing^3–7^, overcomes these problems. Rapid antigen testing has been widely used, and its use has augmented the field of COVID-19 testing. However, the sensitivity of antigen testing is inferior to that of molecular testing^2^, and POCT-type molecular testing is not suitable for the analysis of many samples.

Mobile laboratory systems are alternative options for laboratory systems to manage a large volume of laboratory samples with rapid reporting^8^ and have been clinically implemented during the coronavirus disease (COVID-19) pandemic^9–12^. A hydrogen fuel cell (HFC) bus uses hydrogen as a power source. Water and heat are the only byproducts of HFC vehicles. An HFC bus has a large capacity for energy storage and electricity supply and has the feature of low vibration amplitude for the conversion of hydrogen energy to electric power, both of which are significant merits for mobile clinical laboratories.

**Figure 1:**
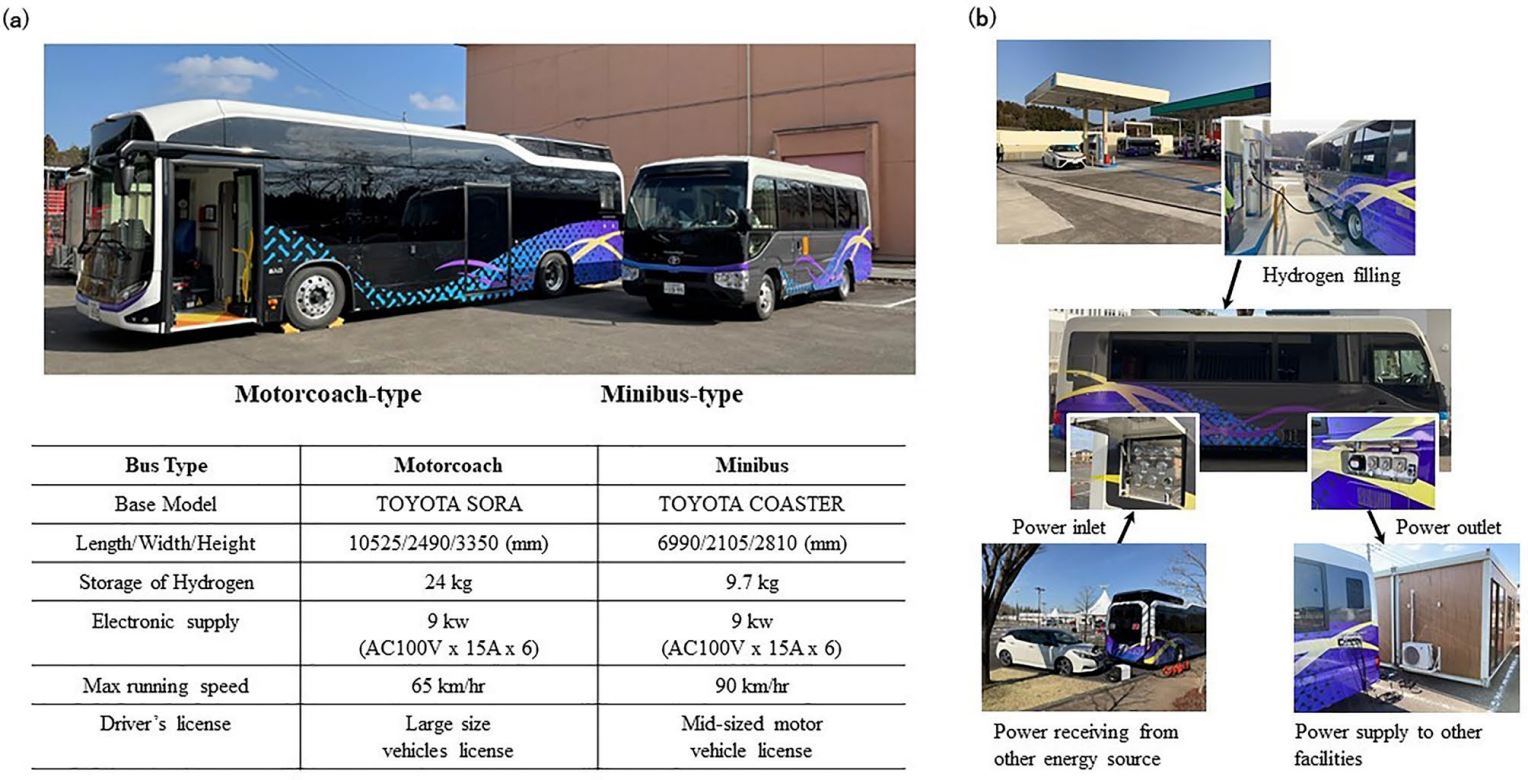
(**a**) Shows general information about two hydrogen fuel cell buses (motorcoach type and minibus type). (**b**) Shows the power supply function and the power receiving function. The logo of the University of Tsukuba and the "Fuel cell Bus" logo on the car has been carefully covered up by the authors.

In 2021, we designed and developed two types of HFC buses equipped with a laboratory system for disasterinfection control (Fig. 1): a motorcoach-type HFC bus (HFC motorcoach) and a minibus-type HFC bus (HFC minibus). The motorcoach was the first commercially available HFC bus in Japan, and was a remodeled SORA (Toyota Motor Corporation). The HFC minibus was fabricated as a prototype vehicle by Toyota Motor Corporation by retrofitting a fuel cell system on a diesel-powered minibus (COASTER; Toyota Motor Corporation), and was remodeled for the current project for use as a mobile laboratory system. Both HFC buses were equipped with automated laboratory systems with electronic reporting, and patients received their laboratory results via e-mail soon after the submission of clinical samples.

In the current study, we evaluated the performance of a mobile laboratory system on HFC buses by focusing on reverse transcription-polymerase chain reaction (RT-PCR) for COVID-19. The mobile range, laboratory capacity, turnaround time (TAT), and accuracy of the laboratory testing were evaluated.

## Methods

This study was performed between October 2021 and March 2022. The evaluation was performed at Tsukuba City Office, Ibaraki Prefectural Office, public health centers, temporary PCR centers, and the University of Tsukuba Hospital. The mobile range was evaluated based on the hydrogen mass consumed during actual driving and RT-PCR testing. The TAT was measured between patient sample submission and the reporting of results via e-mail to each device. The accuracy of laboratory testing was evaluated in comparison with that of a reference RT-PCR assay in a centralized laboratory.

### Specifications of the motorcoach-type and minibus-type HFC buses

Both buses were designed to achieve a maximum electricity output of 9 kW (1.5 kW × 6 outlets, 100 V) for electricity in the laboratory (Fig. 1a) and meet the requirements of biohazard level 2 (P2) according to the World Health Organization (WHO) criteria. Both buses can supply and receive up to 9 kW of electricity (Fig. 1b) with a low noise level during operation (Supplementary Fig. 1). The quality of the power output was confirmed to meet the requirements of medical electrical equipment at ambient temperatures (30 °C/0 °C). Before the current study, both buses were certified to operate on public roads. Both buses permitted only one person as a driver, and no passenger could ride the bus while it was driven. Both the current HFC buses were designed for their equipment, including molecular identification systems, to be changed easily.

**Figure 2:**
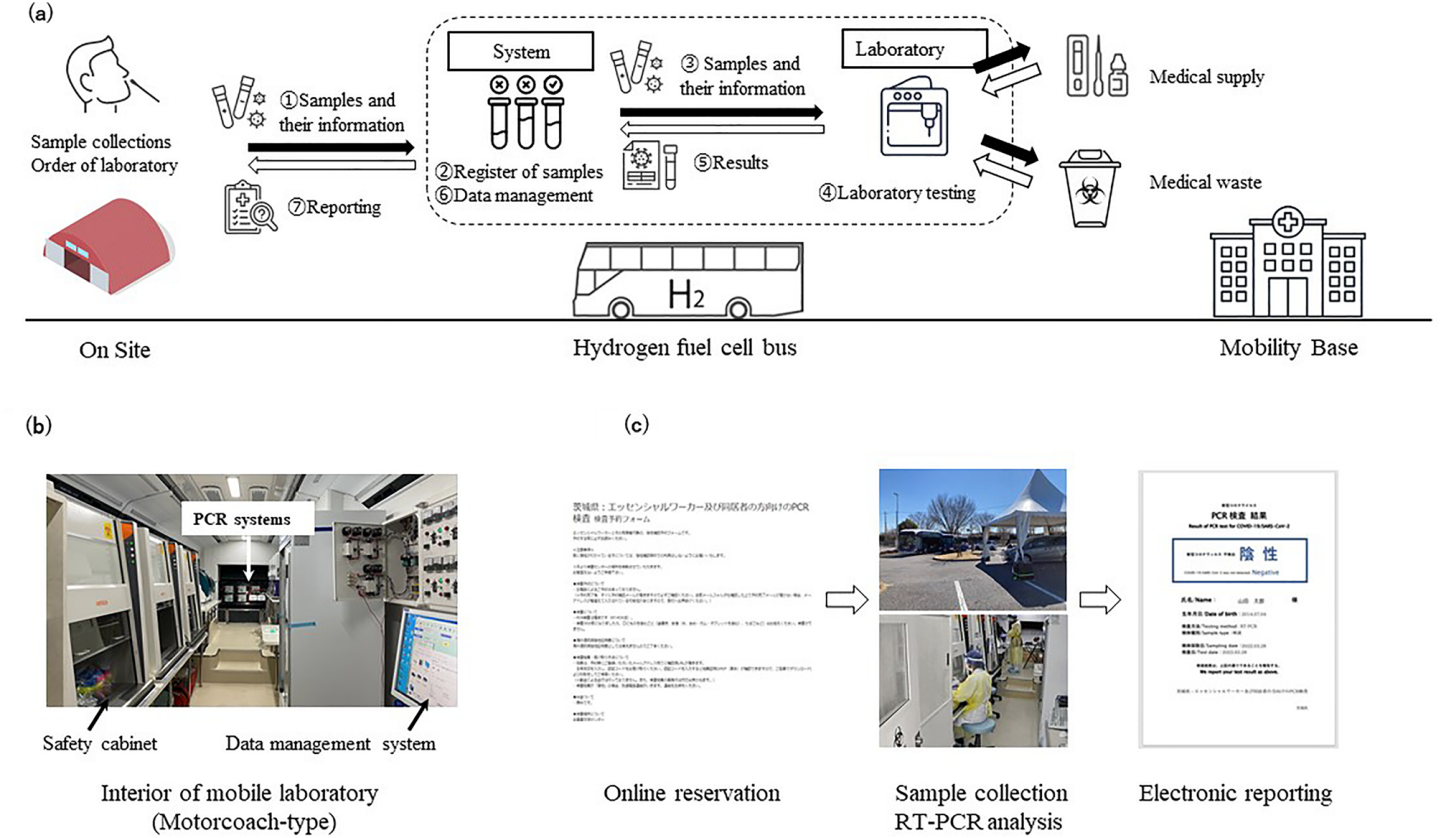
(**a**) Shows the flow of the laboratory system in buses, (**b**) shows a picture of the interior of the motorcoach-type hydrogen fuel cell bus, and (**c**) shows the flow of the electronic reservation and reporting systems.

The general flow of the mobile laboratory system is illustrated in Fig. 2. The laboratory system included an electronic reservation system, a data management system, safety cabinets, refrigerators for samples and reagents, automated purification systems [magLEAD, Precision System Science Co., Ltd. (PSS), Chiba, Japan], automated PCR examination systems (GENECUBE, TOYOBO Co., Ltd., Osaka, Japan)^13–20^, and an electronic reporting system. The Reservations can be performed by the patient using an electronic device, and the laboratory results are sent to their registered e-mail address soon after the submission of the samples. The detailed layout of each bus for laboratory testing is described in Supplementary Fig. 2.

GENECUBE is a Qprobe-PCR-based automated rapid molecular identification system that can detect target genes in a short time and simultaneously analyze up to 24 samples and 4 assays in a single examination. The system automatically performs a direct molecular examination, including preparation of the reaction mixtures and amplification and detection of target genes, in 30 min. GENECUBE HQ SARS-CoV-2 (TOYOBO Co., Ltd.)^13^ has been approved as *In-Vitro* Diagnostics (IVD) product in Japan, and its use along with rapid purification methods with magLEAD^14^, were commercially available in clinical laboratories. Through the examination from preparation of samples to RT-PCR, the entire procedure was performed in the buses using plastic droppers. Positive and negative samples were set to each run in this study, and all positive samples were re-evaluated before reporting. The details of the workflow inside buses and the estimated TAT of each process along with a comparison with a conventional laboratory are summarized in Supplementary Table 1, Supplementary Figs. 3 and 4.

### Hydrogen fuel consumption for driving and laboratory testing (COVID-19 RT-PCR)

Hydrogen fuel consumption by the motorcoach and minibus was measured during a one-way trip to several different testing sites from the University of Tsukuba Hospital, with RT-PCR examinations conducted in those places. Negative anonymized saliva samples and SARS-CoV-2 reference material, the AccuPlex™ SARS-CoV-2 Reference Material Kit (SeraCare; SeraCare Life Sciences, Inc., Milford, Massachusetts, the United States), were used for laboratory testing. The sample size was determined based on the PCR test capacity of each bus (Supplementary Fig. 4).

### Comparison of the diagnostic accuracy of laboratory testing (COVID-19 RT-PCR) in the HFC bus and that of the reference RT-PCR assay in a centralized laboratory

Between February 1, 2022, and March 31, 2022, HFC buses were sent to two temporary COVID-19 PCR centers in Tsukuba at the request of the Ibaraki Prefectural Office (Supplementary Fig. 5). Asymptomatic workers at clinics, hospitals or social welfare facilities who were in close contact with COVID-19 patients in Ibaraki Prefecture used temporary PCR centers. Eligible individuals made an online reservation and submitted saliva samples by the drive-through method in the temporary PCR centers (Fig. 2c). Saliva samples were chosen for the sample of temporary COVID-19 PCR centers due to easier specimen collection compared with nasopharyngeal samples and anterior nasal samples and the nonnecessity of swabs and liquid medium; there are serious shortages of both due to high demand during the COVID-19 pandemic.

The samples were immediately analyzed in the buses, and the results were reported to visitors electronically through their registered email address. We analyzed the data and performed an analysis of the diagnostic accuracy of COVID-19 RT-PCR between February 1, 2022, and February 28, 2022, for the HFC motorcoach and between March 23, 2022, and March 28, 2022, for the HFC minibus. Residual saliva samples were anonymized and preserved at − 80 °C until evaluation.

For the analytical evaluation, the preserved frozen saliva samples were sent to LSI Medience Corporation for comparison, and then a reference real-time RT-PCR examination was performed using a Maxwell® RSC Viral Total Nucleic Acid Purification Kit for ribonucleic acid (RNA) extraction (Promega Corporation, Wisconsin, the United States) according to the manufacturer’s instructions and a Cobas® Z480 Real-Time RT-PCR System (Roche, Basel, Switzerland) using a method developed by the National Institute of Infectious Diseases (NIID), Japan, for SARS-CoV-2^21^. A duplicate analysis for N2 genes was performed for the evaluation of SARS-CoV-2. The Ampdirect 2019 Novel Coronavirus Detection Kit (Shimadzu Corporation, Kyoto, Japan)^22^ and cobas 8800 system and cobas SARS-CoV-2 & Influenza A/B (cobas; Roche Molecular Systems, New Jersey, the United States)^23^ were used to evaluate discrepant samples. The differences in the limit of detection among molecular examinations for COVID-19 are shown in Supplementary Table 2, which was evaluated with SARS-CoV-2 reference material, anonymized nasopharyngeal samples, and saliva samples.

### Ethical considerations

The ethics committee of the University of Tsukuba Hospital approved the protocol of the present study (approval number: R03-043). All methods were performed in accordance with the relevant guidelines and regulations. Written informed consent was obtained from participants who provided saliva samples used for TAT analyses. For other analyses, we used residual samples as a retrospective study that were completely anonymous; therefore, the requirement for obtaining informed consent was waived by the ethics board of the University of Tsukuba Hospital.

### Statistical analyses

The total concordance rate, positive concordance rate, and negative concordance rate were calculated with 95% confidence intervals (CIs). The correlation between the cycle threshold (Ct) value of the real-time RT-PCR assay (NIID method) and the Sp value of GENECUBE was evaluated using Spearman's rank correlation coefficient. P values of < 0.05 were considered to indicate statistical significance. All statistical analyses were conducted using the R 4.1.2 software program (The R Foundation, Vienna, Austria) with the "readxl", "tidyverse", and "epiR" packages.

## Results

### Evaluation of hydrogen fuel consumption in driving and laboratory testing (COVID-19 RT-PCR)

**Figure 3:**
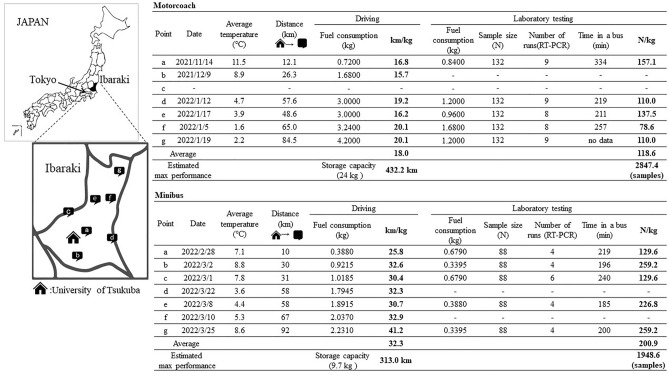
Hydrogen fuel consumption for driving and laboratory testing (COVID-19 RT-PCR). Points a–g in the figure indicate laboratory test sites. a: Tsuchiura Public Health Center, b: Ryugasaki Public Health Center, c: Chikusei Public Health Center, d: Itako Public Health Center, e: Ibaraki Prefectural Office, f: Hitachinaka Public Health Center, g: Hitachi Public Health Center. The maps were created by the authors using Adobe Illustrator ver.28.0. (https://www.adobe.com/). The estimated maximum power of the driving distance and the laboratory testing throughput of COVID-19 RT-PCR of the hydrogen fuel cell bus were calculated according to the hydrogen storage of each bus (motorcoach, 24 kg; minibus, 9.7 kg) and average hydrogen consumption. The difference in driving distance between each bus is due to the driving route of each bus from the University of Tsukuba to the destination. The driving distance was calculated by Google Maps for the motorcoach and by the onboard distance calculation system for the minibus. The data on the average temperature of each site on the examination date were cited from the data on the website of the Japan Meteorological Agency; https://www.jma.go.jp/jma/indexe.html. The temperature inside the buses during the examination was not recorded. RT-PCR, reverse transcription-polymerase chain reaction.

The evaluation of hydrogen fuel consumption in driving and laboratory testing is summarized in Fig. 3. For the motorcoach, the average fuel consumption in driving and laboratory testing (COVID-19 RT-PCR) was 18.0 km/kg (range 15.7–20.1 km/kg) and 118.6 samples/kg (range 78.6–137.5 samples/kg), respectively. For the minibus, the average fuel consumption in driving and laboratory testing (COVID-19 RT-PCR) was 32.3 km/kg (range 25.8–41.2 km/kg) and 200.9 samples/kg (range 129.6–259.2 samples/kg), respectively.

### Analysis of the diagnostic accuracy of COVID-19 RT-PCR in the two HFC buses

During the evaluation period, 18 days of examination data for the motorcoach (n = 1395) and 3 days of examination data (n = 179) for the minibus were evaluated. The median hydrogen consumption during the COVID-19 RT-PCR was 57.2 samples/kg [IQR (interquartile range) 35.2–90.4] for the motorcoach and 135.0 samples/kg (IQR 111.8–177.4) for the minibus (Table 1). For the TAT, the median time between the sample submission and the completion of COVID-19 RT-PCR was 86 min (IQR 73–96 min) for the motorcoach and 76 min (IQR 71–84 min) for the minibus, and the median time between sample submission and electronic reporting of the result to each visitor was 182 min (IQR 134–272 min) for the motorcoach and 194 min (IQR 167–227 min) for the minibus (Table 1). Detailed information is described in Supplementary Table 3.

**Table 1:** Turnaround time (TAT) and hydrogen fuel consumption in COVID-19 PCR centers.

	Temperature (median) (℃)	Median test/day (IQR)	Median TAT (IQR) (min) (Sample collection to patients reporting of the results)	Median TAT (IQR) (min) (Sample collection to completion of evaluation)	Median number of runs (RT-PCR) (IQR)	Median time in bus (IQR) (min)	Median fuel consumption (test/kg) (IQR)
Motorcoach	3.1 (2.5–3.8)	61 (45–120)	182 (134–272)	86 (73–96)	10.5 (9.3–12)	347 (264–358)	57.2 (35.2–90.4)
Minibus	6.6 (5.1–9.5)	64 (54–68)	194 (167–227)	76 (71–84)	8 (7–8.5)	280 (278–288)	135.0 (111.8–177.4)

The average temperature was calculated from the data on the website of the Japan Meteorological Agency (https://www.jma.go.jp/jma/indexe.html). The temperature inside the buses during the examinations was not recorded.

*IQR* interquartile range, *PCR* polymerase chain reaction.

The results of the comparison between the molecular examination in buses and a reference RT-PCR assay in the laboratory are shown in Table 2 (n = 1574). The total concordance rate, positive concordance rate and negative concordance rate were 98.4% (95% CI 97.7–99.0%), 100% (95% CI 95.1–100%) and 98.3% (95% CI 97.6–98.9%), respectively. Of the 25 bus-positive and reference RT-PCR-negative cases, 23 were positive by an Ampdirect 2019 Novel Coronavirus Detection Kit. The 2 samples were further evaluated with a cobas 8800 system and cobas SARS-CoV-2 & influenza A/B, and both samples were found to be positive.

**Table 2:** Comparison between molecular examinations performed in hydrogen fuel cell buses and the reference RT-PCR assay results in the laboratory.

		Real-time RT-PCR in laboratory*	
		Positive	Negative
Molecular examination in bus	Positive	73	25
	Negative	0	1476
Positive concordance rate (%)		100 (95.1–100)	
Negative concordance rate (%)		98.3 (97.6–98.9)	

Data in parentheses indicate 95% confidence intervals.

*RT-PCR* reverse transcription-polymerase chain reaction.

*The real-time RT-PCR procedure for SARS-CoV-2 was developed by the National Institute of Infectious Diseases, Japan^21^.

The correlation of the Sp value of GENECUBE in buses and the Ct value of the reference RT-PCR assay is shown in Supplementary Fig. 6.

## Discussion

To date, this is the first report on the clinical implementation of HFC buses as mobile laboratories. The current evaluation showed that both types of buses had sufficient hydrogen storage for driving and laboratory testing (COVID-19 RT-PCR). With the implementation of the newly developed mobile laboratory systems in COVID-19 PCR centers, visitors could receive the RT-PCR results within a few hours online after sample submission. The high diagnostic accuracy for the detection of SARS-CoV-2 RNA in saliva samples was confirmed by comparison with a reference RT-PCR assay.

The clinical implementation of mobile laboratories has dramatically progressed since the onset of the COVID-19 pandemic. In Japan, the clinical use of mobile laboratories was tentatively approved—as a special case—the COVID-19 pandemic in early February 2022^24^. This approval allowed the current HFC mobile laboratory system to be introduced into clinical practice. Touron et al. reported the utility of the MobilDNA lab for COVID-19^11^ in France. This transportable laboratory system has been available since 2015. The system used was an Applied™ 7500 real-time PCR system operated by highly skilled medical technologists, and it was utilized in the COVID-19 pandemic as a high-throughput mobile laboratory, which reported results in one day. Ballard also reported the rapid reporting of molecular examinations using a mobile laboratory system with an automated molecular identification system (GeneXpert) in Australia^9^. They showed that the median TAT from sample collection to reporting of results was approximately 2 h, which was significantly faster than that of a conventional laboratory; however, it was limited in the number of samples, and the TAT was more than 4 h in some cases.

In our current HFC bus-based mobile laboratory system, laboratory examinations were completed in approximately one hour, most of the results were reported within 3 h after the samples were obtained, and the TAT was improved to 2 h (Supplementary Table 3a) after adjusting for the workflow in late February, which increased the frequency of reporting RT-PCR results from once a day to twice a day for the motorcoach. TAT can be shortened by increasing the frequency of reporting because there is an approximately 100-min interval in both HFC buses between the completion of the molecular examination and the reporting of the results. The test capacity for fuel consumption was clearly lower in the COVID-19 PCR centers (Table 1) than in the initial evaluation (Fig. 3). According to the current evaluation, the capacity was influenced by the average temperature at the test sites and the duration for which the laboratory staff stayed in the HFC buses, both of which are important factors for the consumption of hydrogen for air conditioning. Additionally, the number of tests per run in COVID-19 PCR centers was lower than that in the initial evaluations due to frequent molecular examinations, which shortened the TAT in a real-world clinical setting. Despite these influencing factors, the HFC bus could process 100 samples in temporary COVID-19 PCR centers without a delay in TAT or a hydrogen energy shortage.

In addition, the current system exhibits sufficient analytical performance for the detection of SARS-CoV-2 RNA. Additionally, laboratory processing was automated, including reservation and reporting. All the processes were easy to handle without pipetting during laboratory examination, and the current system can be managed by inexperienced technicians in case of emergency use. The current systems require large amounts of electricity for automated laboratory systems and air conditioning (nearly 9 kW) for the appropriate working environment of technicians, and the HFC bus is essential to supply electricity in addition to the merit of low vibration amplitude.

The current evaluation clarified the characteristics of both HFC buses in a mobile laboratory. The motorcoach has a superior driving range and test capacity and has sufficient workspace for laboratory staff in the bus, whereas the minibus clearly has more efficient hydrogen consumption during driving and laboratory testing. In addition, the minibus can use highway roads and be driven without a special driver’s license. For clinical use, minibus is superior to motorcoaches for rapid response to urgent requirements, such as contact tracing of hospital outbreaks or rapid molecular examination for new emerging infectious diseases at or near airports. In the real-world setting, the minibus was used as a temporary COVID-19 PCR center (14,654 patients) and for contact tracing of COVID-19 outbreaks at 7 healthcare facilities in fiscal year 2022. Meanwhile, the motorcoach will be of use if we need to evaluate a large number of residents per day. Both HFC buses will have significant utility for rapid responses in cases of novel emerging diseases, or for case tracing if highly virulent COVID-19 variants emerge.

Note that the system is not only capable of stand-alone, self-sustaining testing in an HFC bus, but also has the ability to be connected to other energy resources. Future design implications of such mobile laboratories include the ability to supply power to external facilities and receive power from an external source, including mobile sources such as EV or fuel cell vehicles (FCV). In this experiment, when weather conditions made it difficult to travel to the hydrogen station, power could be supplied from the FCV during the inspection, and the inspection could then be performed successfully. It is also important to supply electricity to the surrounding environment, such as a small workspace for sample collection and lighting facilities during the night. We believe that we can connect the developed mobile laboratory to medical institutions and public facilities with safe, robust, and stable mobile testing.

The present study has several limitations. First, the hydrogen fuel consumption during driving and laboratory testing was measured just after the development of the systems, and inexperience may have affected the data. Additionally, the current data were mainly collected in the winter season, which requires heating inside the buses; this requirement differs in other seasons. Second, the current evaluation, including the preceding limit of detection study (Supplementary Table 2), showed sufficient analytical performance of molecular examination for the detection of SARS-CoV-2 RNA; however, we could not use fresh positive samples for comparison with the reference RT-PCR assay. Storage processes involving freezing and thawing have been reported to affect viral load in samples^25^. Third, we used hydrogen energy; however, hydrogen supply and the number of hydrogen fueling stations are clearly insufficient in most countries by 2023, which complicates the universal clinical application of the results of the current research. Third, in the current study, HFC mobile laboratory systems were only evaluated for SARS-CoV-2 by RT-PCR in saliva samples. Current molecular examination platforms can evaluate other respiratory samples and various pathogens^15–20^, and the test platforms of both HFC buses can easily be changed upon request. The feasibility of using other samples, pathogen targets, or other molecular identification platforms for HFC mobile laboratories needs to be evaluated.

In conclusion, the evaluation of the newly developed mobile laboratory system confirmed that HFC buses have sufficient energy storage for driving and laboratory testing (COVID-19 RT-PCR), and that they are feasible for the rapid reporting of highly accurate COVID-19 RT-PCR results.

### Supplementary Information

Supplementary Information.

## Data Availability

The data include sensitive information about the health of human research subjects and thus cannot be directly deposited openly. However, anonymized individual-level data that enable full replication of the study results are available from the corresponding author.
