# Supplementary material for: Development of a mobile laboratory system in hydrogen fuel cell buses and evaluation of the performance for COVID-19 RT-PCR testing
Source: Sci Rep. 2023 Oct 16;13:17546. doi: 10.1038/s41598-023-44925-7 (PMC10579409; doi:10.1038/s41598-023-44925-7)
Supplement: Supplementary file 1 — Supplementary Information. [file 41598_2023_44925_MOESM1_ESM.pdf]

Development of a mobile laboratory system in hydrogen fuel cell buses and evaluation of the performance for COVID-19 RT-PCR testing

Miho Okude[1], Kenji Suzuki[2], Asami Naito[1], Akio Ebashi[3], Tomoka Kusama[3], Junichi Kiyotaki[4,5], Yusaku Akashi[6], Yoshihiko Kiyasu[3,6,7], Yoko Kurihara[3], Shigeyuki Notake[5], Masaki Takanashi[1], Tomokazu Setoyama[1], Yasushi Kawakami[8], Hiromichi Suzuki[3,6*]

1. LSI Medience Corporation, 3-30-1 Shimura, Itabashi, Tokyo 174-8555, Japan
2. Center for Cybernics Research, University of Tsukuba, Tsukuba, 1-1-1 Tennodai, Tsukuba, Ibaraki 305-8573, Japan
3. Department of Infectious Diseases, University of Tsukuba Hospital, 2-1-1 Amakubo, Tsukuba, Ibaraki 305-8576, Japan
4. Miroku Medical Laboratory Inc. 659-2 Innai, Saku, Nagano 384-2201, Japan
5. Department of Clinical Laboratory, Tsukuba Medical Center Hospital, 1-3-1 Amakubo, Tsukuba, Ibaraki 305-8558, Japan
6. Department of Infectious Diseases, Faculty of Medicine, University of Tsukuba, 1-1-1 Tennodai, Tsukuba, Ibaraki 305-8575, Japan
7. Division of Infectious Diseases, Department of Medicine, Tsukuba Medical Center Hospital, 1-3-1 Amakubo Tsukuba, Ibaraki 305-8558, Japan
8. Department of Laboratory Medicine, Faculty of Medicine, University of Tsukuba, 1-1-1 Tennodai, Tsukuba, Ibaraki 305-8575, Japan

* Correspondence to:
Hiromichi Suzuki, MD, PhD
Professor, Department of Infectious Diseases, Faculty of Medicine, University of Tsukuba, 1-1-1 Tennodai, Tsukuba, Ibaraki, 305-8575, Japan
Tel: +81-29-853-3210
E-mail: hsuzuki@md.tsukuba.ac.jp

Supplementary Table 1. A comparison of the approximate turnaround time and hands-on-time between the HFC (hydrogen fuel cell) bus mobile laboratory and a conventional laboratory

Supplementary Table 2. Difference in the limit of detection among molecular examinations for COVID-19.

Supplementary Table 3. Turnaround time (TAT) and hydrogen fuel consumption of the motorcoach-type hydrogen fuel cell bus and minibus type hydrogen fuel cell bus

Supplementary Figure 1. An evaluation of the noise level of the hydrogen fuel cell bus (motorcoach).

Supplementary Figure 2. Detailed layout of the motorcoach-type hydrogen fuel cell bus and minibus-type hydrogen fuel cell bus used for laboratory testing.

Supplementary Figure 3. Details of workflow inside the buses (motorcoach and minibus).

Supplementary Figure 4. Gantt chart of each working process of the hydrogen fuel cell bus mobile laboratory, including the number of laboratory staff and their tasks.

Supplementary Figure 5. Pictures of two temporary COVID-19 PCR centers to which hydrogen fuel cell buses were sent.

Supplementary Figure 6. A comparison between the Sp value of GENECUBE and the cycle threshold (Ct) values of the reference real-time RT-PCR assays (N2 gene).

Supplementary Table 1. A comparison of the approximate turn-around time and hands-on-time between HFC bus mobile laboratory and conventional laboratory

	Molecular examination in HFC buses*	Reference RT-PCR* in a conventional laboratory
Each process	Approximate time** (1 – 48 samples)	Approximate time** (1 – 48 samples)
Transportation of samples into a laboratory from sample collection sites	5 min (3-6 transfer/h)	2 - 4 hours (1 transfer/day)
Sample preparation***	1 – 25	15 – 45
Purification and extraction****	12 – 25	40 – 90
RT-PCR with reagent preparation	25 – 50	145 – 200
Total time	43 – 105	320 – 575
Total hands-on-time including transportation samples into a laboratory	5 – 70	160 – 415
Training period	1-3 days	3 weeks

HFC, hydrogen fuel cell; RT-PCR, reverse transcription-polymerase chain reaction

*The details of the workflow of the molecular examinations performed in HFC buses are described in Supplementary Figure 3. Turnaround time and hands-on-time of Reference RT-PCR in a conventional laboratory are estimated of real-time RT-PCR method developed by the National Institute of Infectious Diseases (NIID method) performed in a conventional laboratory.
**Approximate time of each process was estimated based on the situation of 1 laboratory staff for 1 sample and 2 laboratory staff for 48 samples.

*** For sample preparations, we mixed samples with a vortex mixer and performed 10 seconds of centrifugation for molecular examinations in HFC buses, we mixed samples with a vortex mixer and performed 10 minutes of centrifugation for reference RT-PCR in a conventional laboratory.
****Purification and extraction were performed with a magLEAD, from Precision System Science Co., Ltd. with rapid purification methods [14] for molecular examinations in HFC buses, and with a Maxwell® RSC Viral Total Nucleic Acid Purification Kit for ribonucleic acid (RNA) extraction for reference RT-PCR.

Supplementary Table 2. The differences in the limit of detection among molecular examinations for COVID-19.

Copies/mL	Sample	GENECUBE with magLEAD	RT-PCR (N2 NIID method) with Maxwell	Ampdirect 2019 Novel Coronavirus Detection Kit
		Detection rate (N of detection)		
2500	Total	100% (40/40)	97.5% (39/40)	100% (40/40)
	UTM	100% (8/8)	100% (8/8)	100% (8/8)
	Pooled NP samples	100% (16/16)	93.8% (15/16)	100% (16/16)
	Pooled saliva samples	100% (16/16)	100% (16/16)	100% (16/16)
1000	Total	100% (40/40)	70% (28/40)	100% (40/40)
	UTM	100% (8/8)	87.5% (7/8)	100% (8/8)
	Pooled NP samples	100% (16/16)	81.3% (13/16)	100% (16/16)
	Pooled saliva samples	100% (16/16)	50% (8/16)	100% (16/16)
500	Total	92.5% (37/40)	15% (6/40)	90% (36/40)
	UTM	100% (8/8)	25% (2/8)	100% (8/8)
	Pooled NP samples	93.8% (15/16)	18.8% (3/16)	87.5% (14/16)
	Pooled saliva samples	87.5% (14/16)	6.3% (1/16)	87.5% (14/16)
250	Total	45% (18/40)	15% (6/40)	67.5% (27/40)
	UTM	62.5% (5/8)	12.5% (1/8)	75% (6/8)

	Pooled NP samples	37.5% (6/16)	25% (4/16)	50% (8/16)
	Pooled saliva samples	43.8% (7/16)	6.3% (1/16)	81.3% (13/16)
0	Total	0% (0/40)	0% (0/40)	0% (0/40)
	UTM	0% (0/8)	0% (0/8)	0% (0/8)
	Pooled NP samples	0% (0/16)	0% (0/16)	0% (0/16)
	Pooled saliva samples	0% (0/16)	0% (0/16)	0% (0/16)

RT-PCR, reverse transcription polymerase chain reaction; NP, Nasopharyngeal; UTM, Universal transport medium; NIID, National Institute of Infectious Diseases in Japan

SARS-CoV-2 reference material (AccuPlex™ SARS-CoV-2 Reference Material Kit, SeraCare; SeraCare Life Sciences, Inc., Milford, MA, USA) was diluted with matrix (two UTM™; four pooled negative nasopharyngeal samples and four pooled negative saliva samples) to make 5 difference concentration of samples. The molecular examination with each assay was performed four times for each sample.

Supplementary Table 3. Turnaround time (TAT) and hydrogen fuel consumption of motorcoach type hydrogen fuel cell bus and minibus type hydrogen fuel cell bus

Table 3-a; data of motorcoach

Date	Average temperature (℃)	Sample size (N)	Median TAT (IQR) (min) (Sample collection to patients reporting of the results)	Median TAT (IQR) (min) (Sample collection to completion of evaluation)	Fuel consumption (kg)	Number of run (RT-PCR)	Stay in bus (min)	N/kg
2022/2/1	3.8	28	100 (84-116)	65 (61-65)	1.44	6	244	19.4
2022/2/2	2.5	21	134 (133-140)	62 (62-63)	1.68	7	217	12.5
2022/2/3	3.1	25	135 (110-136)	54 (51-66)	0.96	7	241	26.0
2022/2/4	1.9	26	144 (80-172)	65 (60-77)	0.84	7	264	31.0
2022/2/7	1.9	125	220 (186-311)	89 (81-97)	1.56	14	384	80.1
2022/2/8	2.6	124	255 (186-296)	89 (83-96)	1.8	12	415	68.9
2022/2/9	3	49	270 (161-279)	77 (58-92)	-	10	347	-
2022/2/10	2.5	59	290 (156-302)	85 (66-89)	-	10	358	-
2022/2/14	3.5	169	250 (225-344)	112 (101-116)	1.56	13	421	108.3
2022/2/15	3.8	53	255 (246-266)	80 (74-92)	1.2	9	316	44.2
2022/2/16	5	44	228 (133-257)	72 (62-89)	1.2	10	324	36.7
2022/2/17	3.5	63	275 (238-290)	79 (72-84)	1.32	11	357	47.7
2022/2/18	3.8	81	289 (195-312)	80 (69-89)	0.84	11	no data	96.4
2022/2/21	2.4	144	149 (128-167)	82 (75-92)	1.32	12	360	109.1

2022/2/22	2.5	85	127 (101-139)	90 (84-96)	1.32	13	341	64.4
2022/2/24	2.1	54	184 (168-199)	59 (58-79)	1.08	12	236	50.0
2022/2/25	4.3	106	133 (118-156)	82 (76-95)	1.2	11	347	88.3
2022/2/28	6.4	139	135 (118-160)	89 (80-95)	1.32	10	349	105.3

Table 3-b; data of minibus

Date	Average temperature (℃)	Sample size (N)	Median TAT (IQR) (min) (Sample collection to patients reporting of the results)	Median TAT (IQR) (min) (Sample collection to completion of PCR evaluation)	Fuel consumption (kg)	Number of run (RT-PCR)	Stay in bus (min)	N/kg
2022/3/23	3.5	64	229 (211-249)	82 (68-93)	0.29	9	295	219.9
2022/3/24	6.6	43	169 (156-190)	72 (71-84)	0.49	6	275	88.7
2022/3/28	12.3	72	175 (147-216)	76 (71-84)	0.53	8	280	135.0

IQR; interquartile range

We increased the frequency of reporting RT-PCR results from once a day to twice a day from February 21 for the motorcoach; this was not applied for minibus. In February 24, reporting was performed only one time due to the smaller sample size in RT-PCR examinations.

Supplementary Figure 1. The evaluation of noise level of the hydrogen fuel cell bus (motorcoach). Noise levels were measured at six points inside and around the hydrogen fuel cell bus during operation and non-operation periods. The logo of the University of Tsukuba and the "Fuel cell Bus" logo on the car has been carefully covered up by the authors.

Point	M1	M2	M3	M4	M5	M6
Noise level of hydrogen fuel cell bus (dB)	51.6	60.4	54.7	57.4	50.4	73.6
Noise level of background environment (dB)	43.2	43.7	42.3	42.1	42.8	22.9

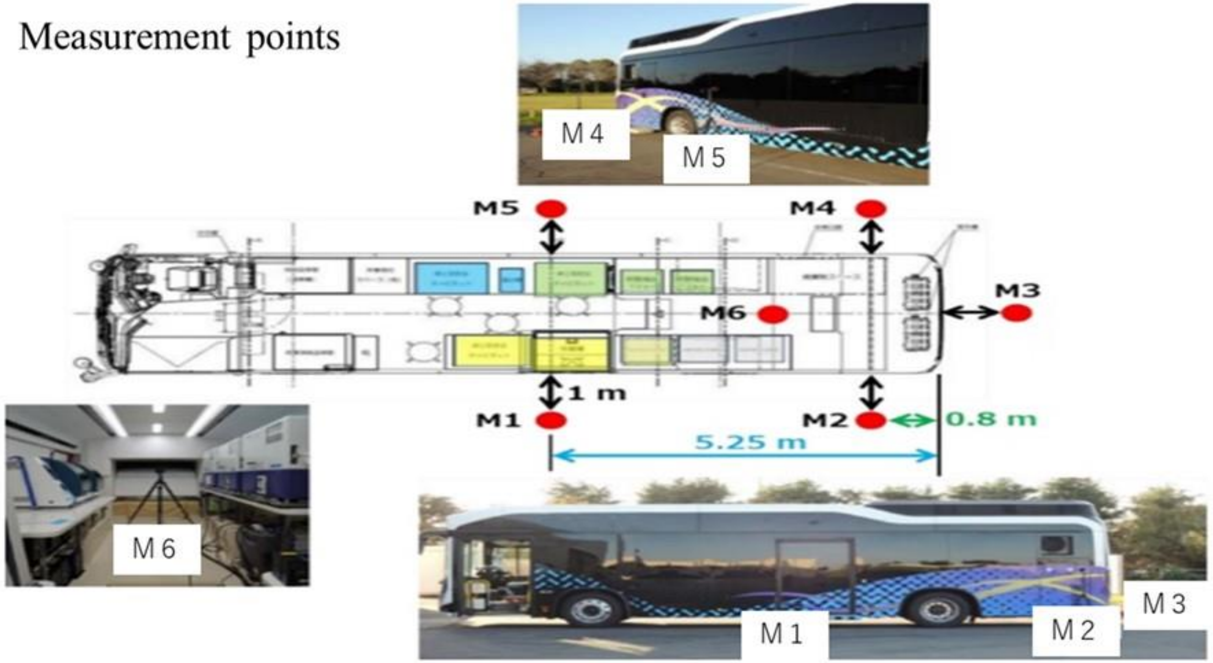

Supplementary Figure 2. Detailed layout of the motorcoach type hydrogen fuel cell bus and minibus type hydrogen fuel cell bus used for laboratory testing.

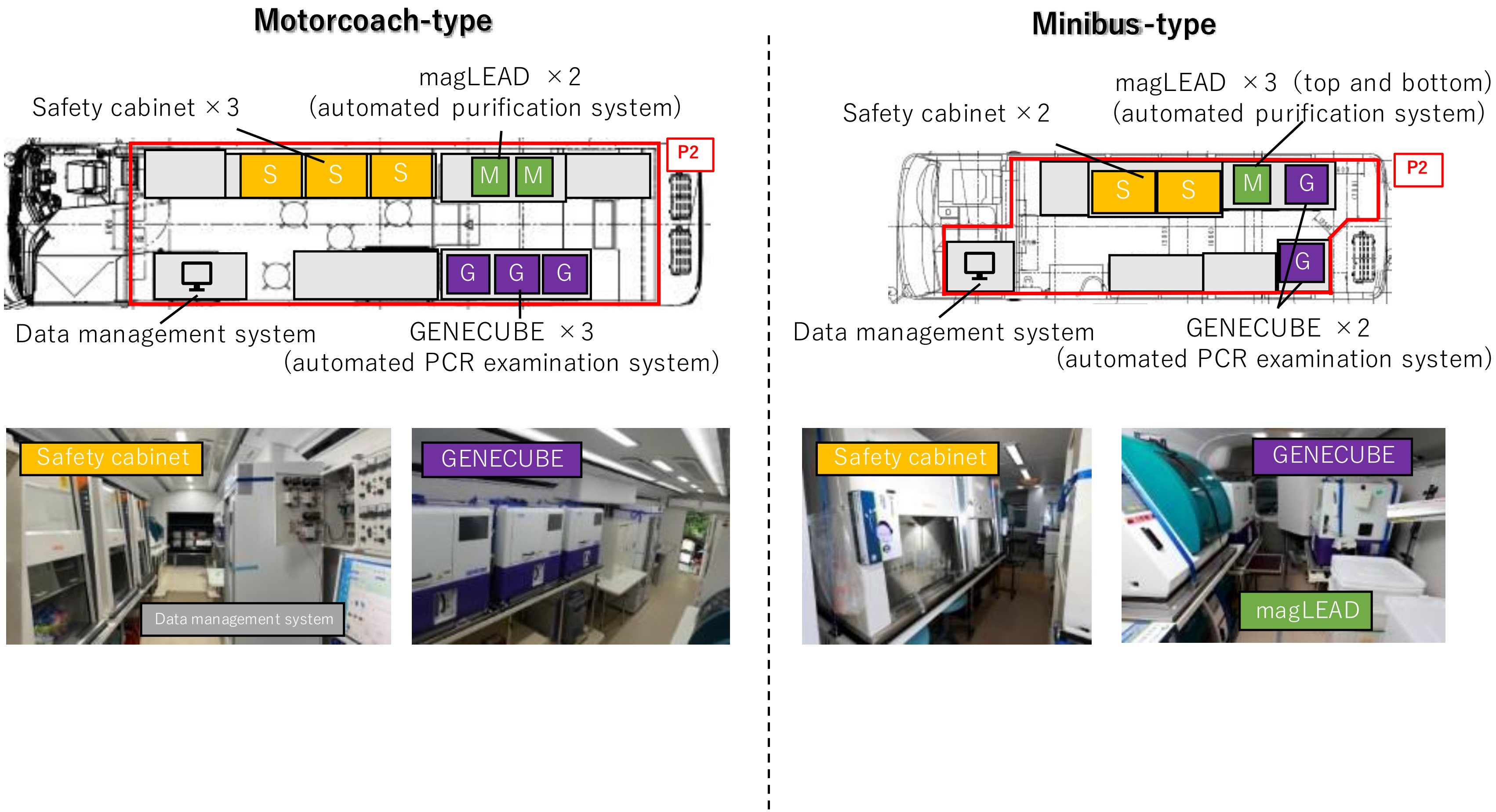

Supplementary Figure 3. Details of workflow inside the buses (motorcoach and minibus).

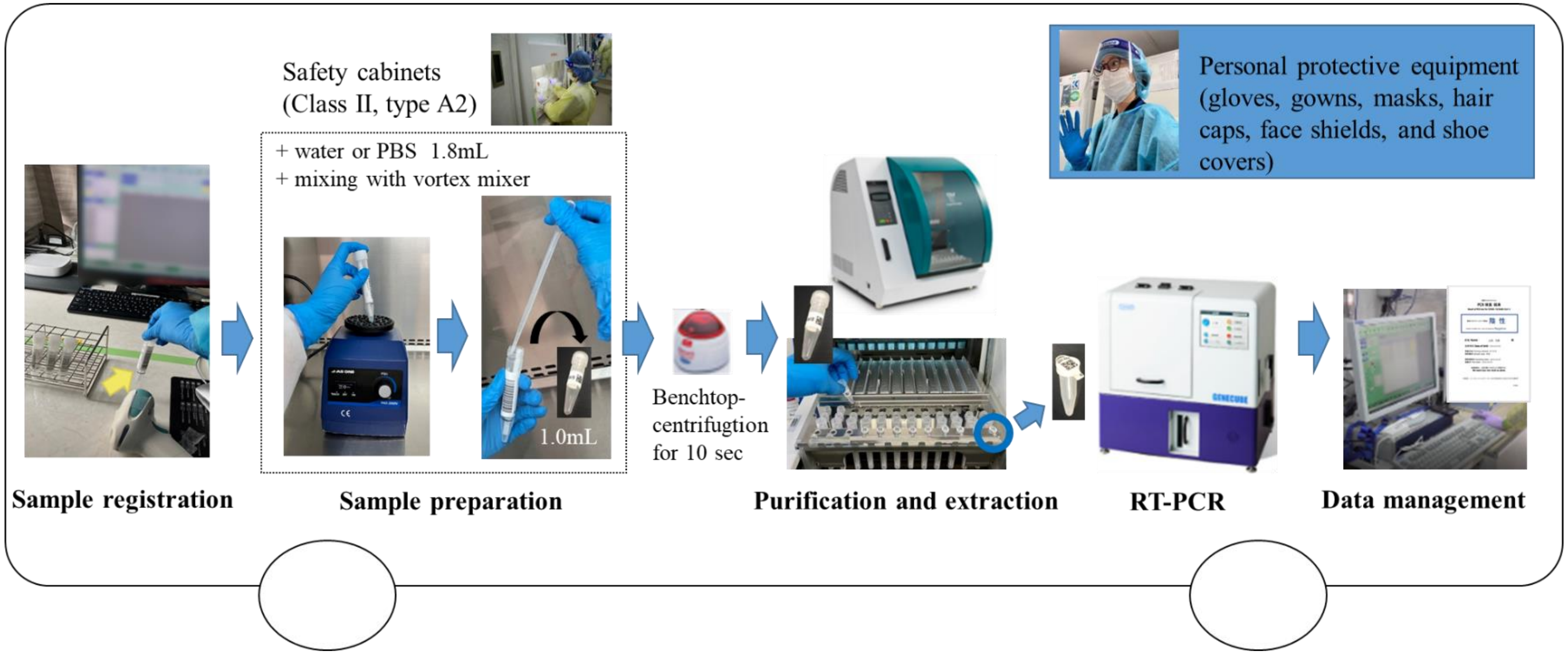

The recommended volume of saliva samples for submission to laboratory in buses was approximately 1 mL. This was the volume that was instructed to be obtained for each patient (black line in saliva tube, which is shown in the picture of sample registration with a yellow arrow). After sample registration, 1.8 mL of water or phosphate-buffered saline (PBS), which was prepared in advance before examination, was added to each sample and the samples were stirred with a vortex mixer for approximately 30 seconds. After mixing, 1 mL of mixed sample was put into a 1.5 mL tube with a plastic dropper with centrifugation for approximately 10 seconds with a small desktop centrifuge. The preparation process was performed in a safety cabinet (Class II, type A2; BHC-T701IIA2, AIRTECH JAPAN, LTD., Tokyo, Japan) wearing personal protective equipment worn (including gloves, gown, surgical mask, hair cap, face shield, and shoe covers). After the preparation process, purification, and ribonucleic acid (RNA) extraction were performed with magLEAD (Precision System Science Co., Ltd., Chiba, Japan) with 200 µL of prepared saliva samples. RNA was eluted in 100 µL, which was used for reverse transcription polymerase chain reaction (RT-PCR) examination with the GENECUBE (TOYOBO Co., Ltd., Osaka, Japan).

Supplementary Figure 4. Gantt chart of each working process for the mobile laboratory with hydrogen fuel cell buses, including the number and tasks of the laboratory staff.

Process		Number of laboratory staff members: Motorcoach (max 5)	Number of laboratory staff members: Minibus (max 3)	Frequency or capacity	Approximate time for each process: each process included preparation and cleaning process
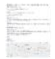	Online reservation			Each patient	
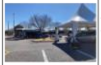	Patient's arrival (patient identification)			Each patient	5-15 min
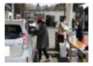	Sample collection and submission			Each patient	15-30 min
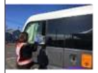	Transfer samples to buses			3 - 6 transfer/hr	5 min
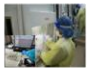	Sample registration and sample preparation	1-3 staff	1-2 staff	Each sample	1 - 25 min
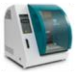	Purification and RNA extraction	1-2 staff	1-2 staff	1 - 24 samples/run	12 - 25 min
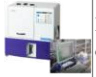	RT-PCR and data check			1 - 72 (motorcoach) or 48 (minibus) samples/run	25 - 50 min
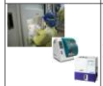	Re-examination for positive or invalid samples	As above		1- 2 runs/day	38 - 50 min
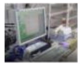	Report RT-PCR results	1 staff (sample registration staff)		Once or twice/day	5 - 10 min
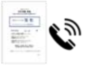	Medical history taking (COVID-19 patients)			Each COVID-19 patient	10 - 60 min

Supplementary Figure 5. Pictures of two temporary COVID-19 PCR centers, to which hydrogen fuel cell buses were sent. The logo of the University of Tsukuba and the "Fuel cell Bus" logo on the car has been carefully covered up by the authors.

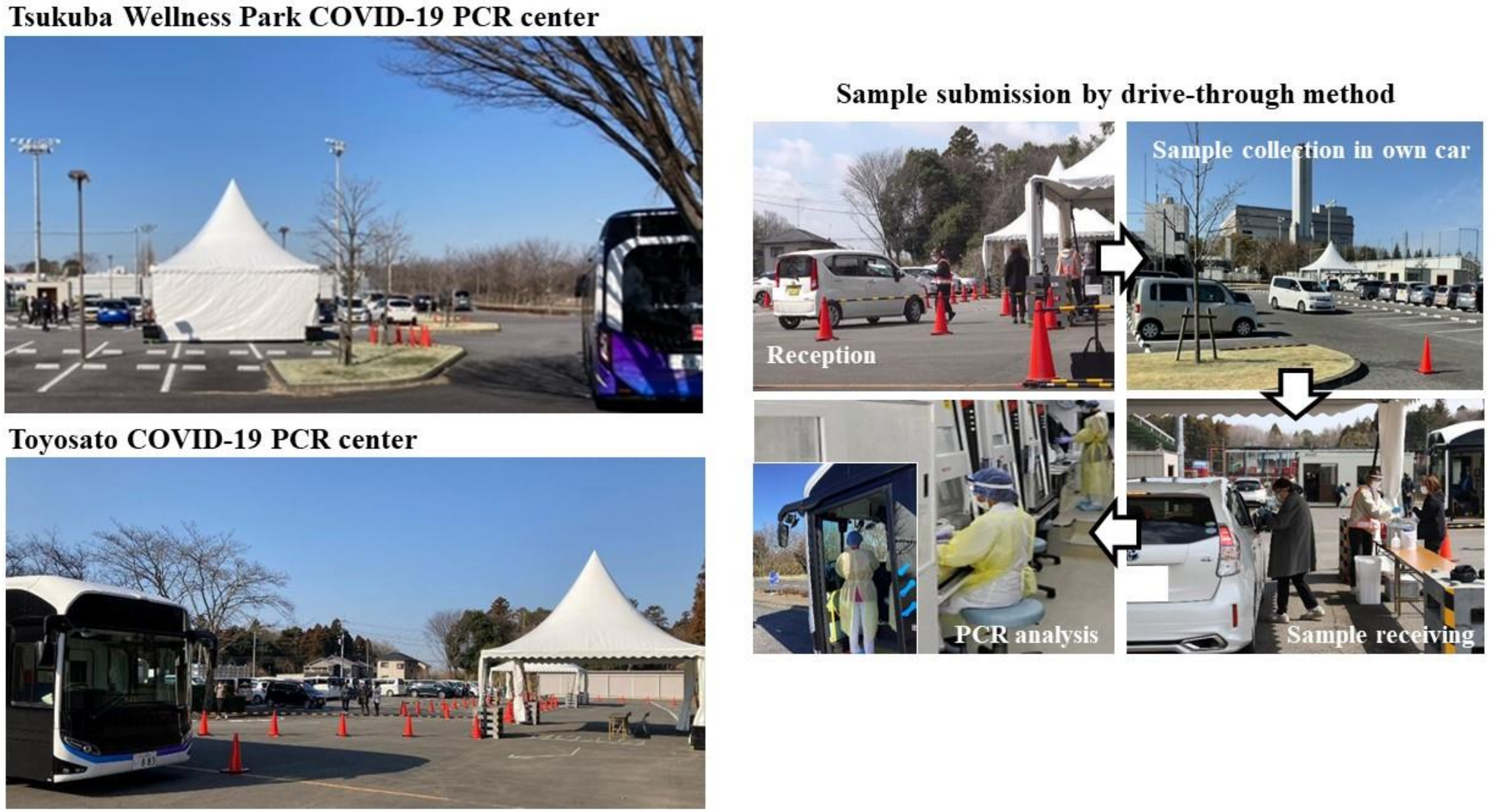

Supplementary Figure 6. Comparison between the Sp value of GENECUBE and cycle threshold (Ct) values of reference real-time RT-PCR assays (N2 gene).

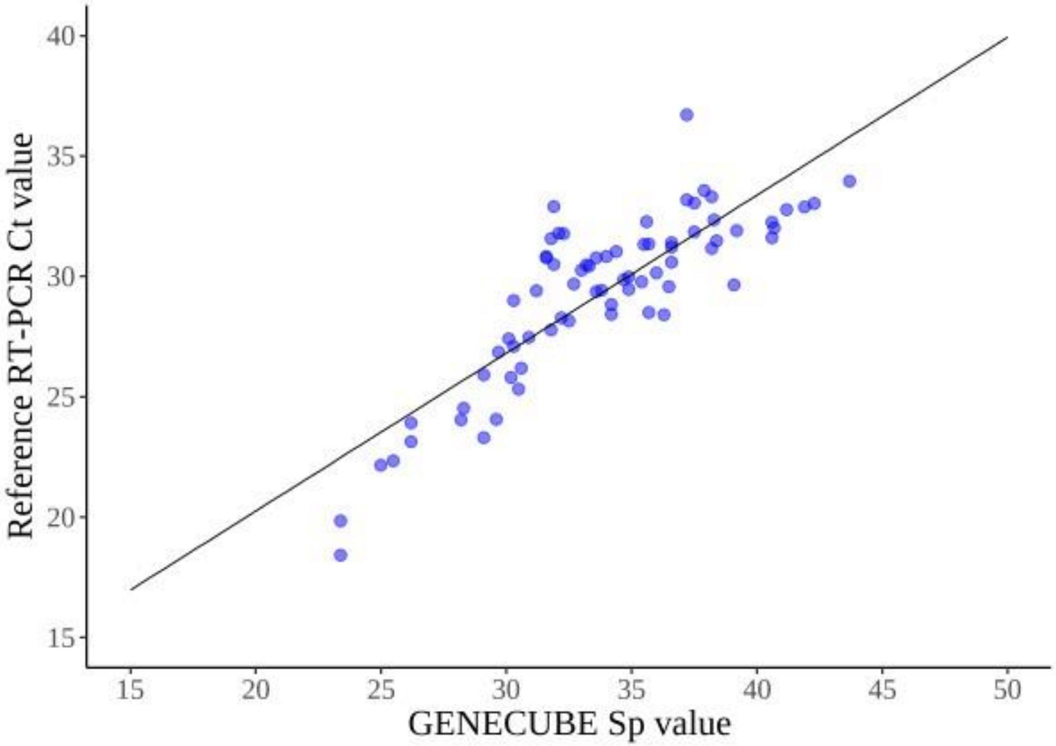

Spearman's rank correlation coefficient (R) between the two tests was 0.81. The 25 GENECUBE-positive and reference RT-PCR-negative cases are not included. The median Sp value was 34.9 (IQR:31.8–38.2) for GENECUBE and the median Ct value was 30.1 (IQR:27.8–31.6) for the reference real-time RT-PCR assay. The median difference was 4.5 (IQR:3.0–5.8).
